# More Effective Mobilization of Hg^2+^ from Human Serum Albumin Compared to Cd^2+^ by L-Cysteine at Near-Physiological Conditions

**DOI:** 10.3390/toxics11070599

**Published:** 2023-07-08

**Authors:** Astha Gautam, Jürgen Gailer

**Affiliations:** Department of Chemistry, 2500 University Drive NW, Calgary, AB T2N 1N4, Canada; agautam@ucalgary.ca

**Keywords:** cadmium, mercury, toxicological chemistry, bloodstream, organ uptake, nephrotoxicity

## Abstract

Although chronic low-level exposure to Hg^2+^ and Cd^2+^ causes human nephrotoxicity, the bioinorganic processes that deliver them to their target organs are poorly understood. Since the plasma protein human serum albumin (HSA) has distinct binding sites for these metal ions, we wanted to gain insight into these translocation processes and have employed size-exclusion chromatography coupled on-line to an inductively coupled plasma atomic emission spectrometer using phosphate-buffered saline mobile phases. When HSA ‘labeled’ with Hg^2+^ and Cd^2+^ (1:0.1:0.1) using 300 μM of L-methionine was analyzed, the co-elution of a single C, S, Cd, and Hg peak was observed, which implied the intact bis-metalated HSA complex. Since human plasma contains small molecular weight thiols and sulfur-containing metabolites, we analyzed the bis-metalated HSA complex with mobile phases containing 50–200 µM of L-cysteine (Cys), D,L-homocysteine (hCys), or glutathione (GSH), which provided insight into the comparative mobilization of each metal from their respective binding sites on HSA. Interestingly, 50 µM Cys, hCys, or GSH mobilized Hg^2+^ from its HSA binding site but only partially mobilized Cd^2+^ from its binding site. Since these findings were obtained at conditions simulating near-physiological conditions of plasma, they provide a feasible explanation for the higher ‘mobility’ of Hg^2+^ and its concomitant interaction with mammalian target organs compared to Cd^2+^. Furthermore, 50 µM Cys resulted in the co-elution of similar-sized Hg and Cd species, which provides a biomolecular explanation for the nephrotoxicity of Hg^2+^ and Cd^2+^.

## 1. Introduction

Since approximately 9 million people globally die of pollution-related causes every year, the exposure of human populations to pollutants has become an imminent public health concern [1]. One important emission source of pollutants is the manufacturing of consumer goods, which requires building blocks (i.e., chemical elements) and the consumption of unprecedented amounts of toxic metal(loid)-laden fossil fuels for their assembly. In fact, 35–77% of anthropogenic mercury emissions are attributed to the combustion of fossil fuels and wastes [2]. Owing to the exceptional longevity of mercury and other toxic metals in the environment [3], the chronic exposure of certain human populations, including children, to these in many parts of the world is concerning [4,5], especially since it is known that exposure to exceedingly small daily doses of certain toxic metal species can severely affect human health [6,7]. The urgent need to establish causal relationships between chronic human exposure to toxic metal species and certain diseases thus requires insight into the biomolecular mechanisms that govern the exposure–response relationship [8].

From a purely bioinorganic chemistry point of view, the metabolism of toxic metal species in the bloodstream is important to understand organ-based adverse effects [8], but it is rather poorly understood because of the underlying biological complexity [9]. Conceptually, we need to distinguish between two types of bioinorganic processes that are toxicologically relevant. The first type refers to toxic metal species that interact with essential trace elements and decrease their organ availability [10] and/or biomolecules within red blood cells, which may adversely affect the organs downstream over time. With regard to the latter, human exposure to Hg^2+^, CH_3_Hg^+^, and CH_3_CH_2_Hg^+^ (a breakdown product of the anti-bactericidal additive thimerosal) will result in the formation of hemoglobin (Hb) complexes within red blood cells [11], which in the case of CH_3_CH_2_Hg^+^ will adversely affect the binding of O_2_ to Hb [12]. The second type refers to mechanisms that mediate the transport of a toxic metal species by plasma proteins to its target organs, followed by the actual toxicological mechanism of action itself unfolding therein. An example of the latter mechanism is the involvement of small molecular weight (SMW) thiols, which are present in blood plasma, in the delivery of the neurotoxin CH_3_Hg^+^ to the blood–brain barrier (BBB) [13].

In blood, the most abundant protein, which comprises more than half of the total protein in plasma is human serum albumin (HSA, 66.3 kDa, ~36–54 g/L) [14], which serves as the transport protein for drugs [15], vitamins, hormones, and numerous metal ions. [16] Owing to its extraordinary ligand-binding properties, HSA is considered to be the most important metal transporter in the bloodstream [17]. HSA has at least two distinct binding sites for Cd^2+^ [18,19], but only the strong one has been structurally characterized [18]. Hg^2+^ has been hypothesized to bind to Cys-34 in HSA [20], but this was experimentally confirmed only recently [21]. HSA is therefore likely to be directly implicated in the transport of both of these toxic metal ions to their respective toxicological target organs [17,22].

Another important class of biomolecules that are implicated in the blood-based delivery of toxic metal species to target organs are the SMW thiols Cys and hCys [23], which are present in the plasma of healthy adults at concentrations up to 9.2 µM [24] and 0.65–12 µM [24,25], respectively. In certain disease states, such as hyperhomocysteinemia, for example, the plasma concentration of hCys can reach up to 500 µM [26]. From a toxicological point of view, the formation of (Cys)_2_Hg complexes under near-physiological conditions [27] is known to play a key role in the translocation of Hg^2+^ to target organs such as the kidneys [23,28,29]. Similarly, Cd^2+^ can form complexes with Cys, including [CdCys]^+^, [Cd(Cys)_2_], [Cd(Cys)_3_]^-^, and [Cd_2_(Cys)_3_]^+^ [30], some of which may also form at physiological pH [31,32]. Although Cys and hCys are likely involved in the uptake of Hg^2+^ from the bloodstream into the kidneys [23], the involved bioinorganic processes that mediate its mobilization from HSA to Hg/Cys and/or Hg/hCys complexes that are then absorbed by the kidneys have not been reported.

To gain insight into the role that Cys and hCys may play in the uptake of toxic metal species from HSA to uptake mechanisms that are located at the surface of target organs, Hg^2+^ and Cd^2+^ were added to pure HSA to obtain an HSA/Hg/Cd complex with a molar ratio of 1.0:0.1:0.1. This molar ratio was chosen to predominantly occupy the strong binding sites on HSA and to observe sufficiently intense Hg/Cd signals in the chromatograms. The latter complex was then analyzed using an established bioanalytical method that comprised size-exclusion chromatography (SEC) coupled on-line to an inductively coupled plasma atomic emission spectrometer (ICP-AES) using phosphate-buffered saline (PBS) buffer as the mobile phase [33]. The application of SEC–ICP–AES allowed us to observe the elution of the HSA/Hg/Cd complex by its elemental signature at near-physiological conditions. Thereafter, increasing physiologically relevant concentrations of Cys and hCys were added to the mobile phase, and the HSA/Hg/Cd complex was analyzed. The unique capability of the employed bioanalytical method to simultaneously observe Cd and Hg in the column effluent allowed us to observe the dose-dependent effect of Cys and hCys on the integrity of the HSA/Hg/Cd complex. While our results are of limited clinical relevance, they are of toxicological importance as HSA-bound Hg^2+^ and Cd^2+^ are inherently less dangerous to organs compared to the SMW-bound complexes of these metal species, which exhibit high organ availability and, therefore, toxicity [13,23]. Our results therefore only provide insight into which Hg/Cd species likely impinge on toxicological target organs but not on the toxicological effects of Hg^2+^ within organs [34], which can be decreased by the long-term administration of an algae extract to patients who are suffering from the release of Hg^2+^ from dental implants and amalgam fillings [35].

## 2. Materials and Methods

### 2.1. Chemicals and Solutions

For this study, 0.01 M phosphate-buffered saline powder sachets (0.01 M NaH_2_PO_4_, 0.138 M NaCl, 0.0027 M KCl, pH 7.4), L-cysteine (Cys, ≥98% purity), D, L-homocysteine (hCys, ≥95% purity), L-glutathione (>98%), L-methionine (Met, >98%), HgCl_2_ (99.5%), and CdCl_2_ (99.99%) were purchased from Sigma-Aldrich (St. Louis, MO, USA). A solution of gel-filtration standards (bovine thyroglobulin—670 kDa, bovine γ-globulin—158 kDa, chicken ovalbumin—44 kDa, horse myoglobulin—17.5 kDa, and vitamin B_12_—1.35 kDa) was purchased from Bio-Rad Laboratories (Hercules, CA, USA). An aqueous solution of human serum albumin (HSA, 25%) in an aqueous diluent (0.08 mmol of sodium caprylate and 0.08 mmol of acetyltryptophan per gram of albumin) was obtained from Grifols Therapeutics LLC (Research Triangle Park, NC, 27709 USA; LOT C5ALF00463). Deionized (DI) water from a Simplicity UV water purification system (Millipore, Billerica, MA, USA) was used to make all solutions.

### 2.2. ‘Labeling’ of HSA with Hg^2+^ and Cd^2+^

A total of 0.8 mL of the stock HSA solution was quantitatively transferred to a 5.0 mL volumetric flask and filled to the mark with PBS buffer (pH 7.4) to obtain a pale-yellow solution of 40 g HSA/L, which was freshly prepared daily prior to analysis. HgCl_2_ (0.1635 g) was weighed into a 10.0 mL volumetric flask and filled to the mark with DI water, and 2 mL of this solution was filled to the mark in a 10.0 mL volumetric flask. This HgCl_2_ solution (3.27 g HgCl_2_/L) was kept in the fridge until use. CdCl_2_ (0.1105 g) was weighed into a 10.0 mL volumetric flask and filled to the mark with dI water, and 2 mL of this solution was filled to the mark in a 10.0 mL volumetric flask. This CdCl_2_ solution (2.21 g CdCl_2_/L) was kept in the fridge until use. To make an aqueous solution that contained HSA, Hg^2+^, and Cd^2+^ at a molar ratio of 1.0:0.1:0.1, an aliquot of the 40 g/L HSA solution (2 mL) was transferred to a polypropylene vial, and 10 µL of the HgCl_2_ solution was added. After gentle agitation, 10 µL of the CdCl_2_ solution was added. Following gentle agitation, the obtained HSA/Cd/Hg complex was filtered using a 0.45 µm Millex-Hv low-protein-binding PVDF membrane syringe filter (Merck Millipore, Ltd., Tullagreen, Carrigtwohill, Cork, Ireland) prior to analysis. The HSA/Cd/Hg complex was made fresh daily for the execution of the experiments outlined below.

### 2.3. Sample Preparation

PBS buffer was prepared by dissolving a sachet in DI water and filling it to the 1.0 L mark. To prepare the mobile phases, which contained SMW sulfur compounds, N_2_(g) was bubbled through the PBS buffer for 60 min to reduce the amount of dissolved O_2_ and preclude the oxidation of the thiols, and then the appropriate amount of a given thiol was added. Thereafter, the pH was adjusted with 4.0 M NaOH to pH 7.4 using a VWR Symphony SB20 pH meter (Thermo Electron Corporation, Beverly, MA, USA). All mobile phases were filtered through a 0.45 µm pore size MF-Millipore MCE membrane filter (Merck Millipore Ltd. Tullagreen, Cartwrightwohill, Co, Cork, Ireland).

### 2.4. Instrumentation

The SEC-ICP-AES system was comprised of an Agilent 1200 series binary SL HPLC pump and a Rheodyne 9010 injector equipped with a 500 µL sample loop. A Superdex^TM^ 200 Increase 10/300 GL high-resolution SEC column (8 µm particle size, fractionation range: 600–10 kDa; GE Healthcare, USA) was used at a flow rate of 1.00 mL min^−1^ using a PBS-buffer mobile phase that contained various concentrations of Cys, hCys, GSH, or Met. All separations were conducted at room temperature (20 °C). Simultaneous multi-element specific detection of C (193.091 nm), S (180.731 nm), Cd (226.502 nm), and Hg (253.652 nm) in the column effluent was achieved with a Prodigy High-Dispersion radial-view ICP-AES (Teledyne Leeman Labs, Hudson, NH, USA) using a radio frequency (RF) power of 1.3 kW, an Ar gas coolant flow rate of 19 L min^−1^, an auxiliary flow rate of 0.5 L min^−1^, and a nebulizer gas pressure of 25 psi. A 240 s delay was implemented between injection and data acquisition. Data were collected for 25 min, unless otherwise stated. The raw data were collected using the manufacturer’s software (SALSA), imported into Sigma Plot 14.5, and smoothed using the bisquare algorithm. Peak areas and retention times were determined using Origin software (Version 2020 b). All experiments were performed in triplicate, allowing the calculation of standard deviations. The size-calibration molecular-weight (MW) markers are depicted on top of all figures to facilitate the discussion of the obtained results. We note that the Hg- and Cd-specific chromatograms with the various mobile phases were obtained simultaneously from one injection of the HSA/Hg/Cd complex.

## 3. Results

We investigated the effect of physiologically relevant Cys and hCys concentrations dissolved in the PBS-buffer mobile phase on the stability of an HSA/Hg/Cd complex [13,36,37]. It is important to utilize PBS buffer in the execution of these studies because this buffer closely resembles the chemical composition of human blood plasma and therefore precludes the generation of artefacts, which have been observed when plasma metalloproteins were previously analyzed using different biochemical buffers [38].

**Figure 1:**
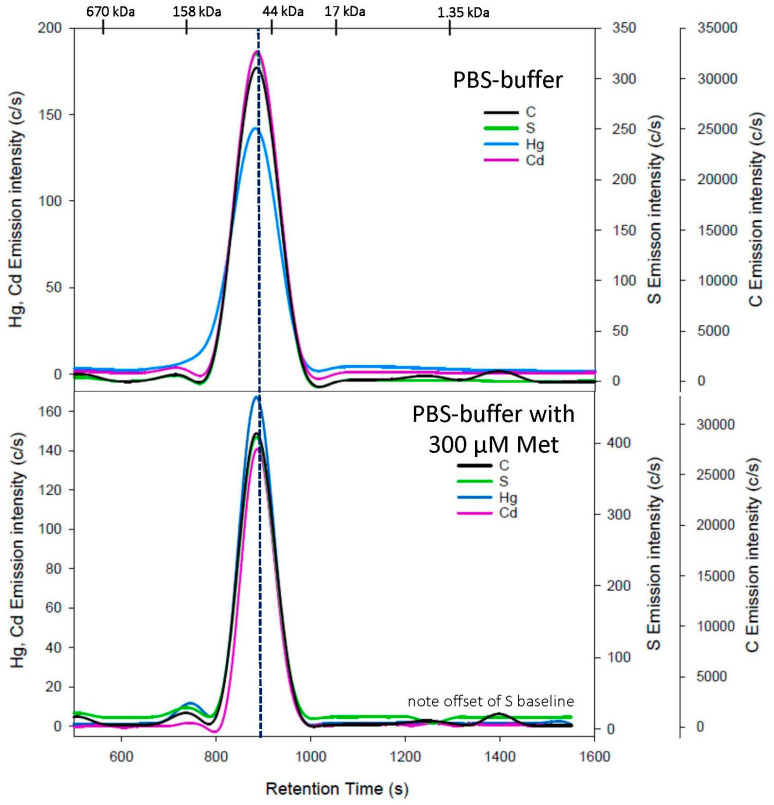
Representative C-, S-, Hg-, and Cd-specific chromatograms obtained using SEC-ICP-AES for the analysis of an HSA/Hg/Cd complex with a PBS-buffer mobile phase (**top**) and PBS buffer containing 300 µM L-methionine (**bottom**) at pH 7.4 using a Superdex 200 Increase 10/300 GL SEC column (10 × 300 mm ID × length, 8 µm particle size, fractionation range: 600–10 kDa). Flow rate: 1.0 mL/min; injection volume: 500 µL; detector: Prodigy High-Dispersion ICP-AES multi-element detector of C at 193.091 nm, S at 180.731 nm, Hg at 253.652 nm, and Cd at 226.502 nm. Retention times of molecular weight standards are indicated at the top.

The analysis of an HSA/Hg/Cd complex with PBS buffer resulted in a single Hg peak (t_r_ = 880 ± 2 s) and a single Cd peak (t_r_ = 887 ± 2 s), as well as co-eluting single C and S peaks (Figure 1, top). The co-elution of Hg and Cd shortly before the 44 kDa MW marker is congruent with the elution of the intact HSA/Hg/Cd complex. When the same HSA/Hg/Cd complex was analyzed with a PBS buffer that contained 300 µM Met, essentially identical Hg-, Cd-, C-, and S-specific chromatograms were observed (Figure 1, bottom), which indicates that Met—which does not contain a free thiol group—did not adversely affect the integrity of the HSA/Hg/Cd complex. We note that one major limitation of our chosen approach is the fact that it does not allow us to investigate <50 µM of Cys or hCys in the mobile phase, as these are prone to rapid oxidation at neutral pH [13]. To enhance the clarity of the discussion that follows, we will now discuss the obtained results separately for each SMW thiol and each metal.

### 3.1. Cys Mobile Phase

**Figure 2:**
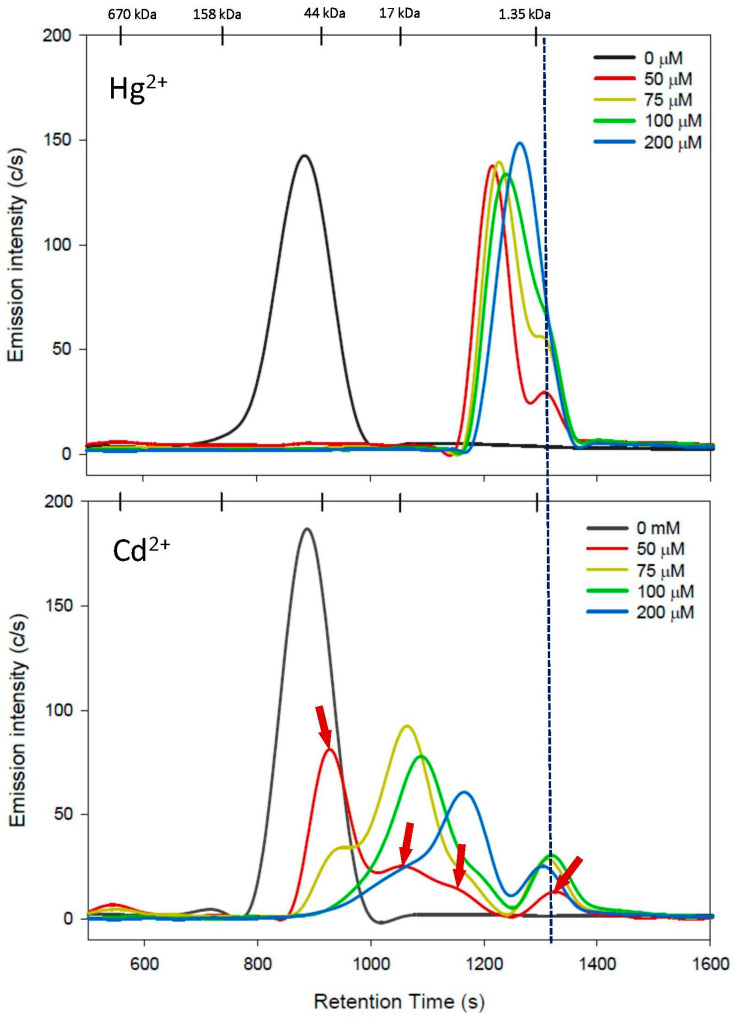
Representative Hg-specific (**top**) and Cd-specific (**bottom**) chromatograms obtained using SEC-ICP-AES for the analysis of an HSA/Hg/Cd complex with a mobile phase containing Cys (from 0 to 100 µM) in PBS buffer (pH 7.4) using a Superdex 200 Increase 10/300 GL SEC column (10 × 300 mm ID × length, 8 µm particle size, fractionation range: 600–10 kDa). Flow rate: 1.0 mL/min; injection volume: 500 µL; detector: Prodigy High-Dispersion ICP-AES of Hg at 253.652 nm and Cd at 226.502 nm. Retention times of molecular weight standards are indicated at the top. The vertical line refers to the smallest peak that was detected for Hg and Cd.

The effect of increasing Cys mobile-phase concentrations on the stability of an HSA/Hg/Cd complex is depicted in Figure 2 and the retention times of the corresponding Hg and Cd peaks are shown in Table 1. The total Hg and Cd peak areas that were obtained with increasing Cys mobile-phase concentrations (50–200 µM Cys) were compared to those obtained for the Cys-free mobile phase, which revealed Hg and Cd recoveries of 66–73% and 51–75%, respectively (Table 1). These recoveries can be rationalized by the partial mobilization of Hg and Cd from HSA to thiol complexes, some of which may have reacted with other mobile-phase constituents (e.g., HPO_4_^2−^) the reaction products of which subsequently adsorbed onto the stationary phase.

With the 50 µM Cys mobile phase, a large Hg peak eluted first (t_r_ = 1210 ± 3 s), followed by a non-baseline-separated smaller Hg peak on its long retention end (t_r_ = 1304 ± 2 s) (Figure 2, top). The elution of both peaks close to the inclusion volume (see the dotted line close to the 1.35 kDa MW marker) implies that Hg^2+^ was mobilized from HSA and eluted in the form of two distinct Hg complexes. Based on previous studies by others, the first Hg peak is tentatively identified as a mixture of Hg_2_Cys_2_, Hg_3_Cys_2_, Hg_2_Cys_2_HCl, Hg_3_Cys_2_Cl_2_, and Hg_3_Cys_2_Cl_6_ ^.^ 2H_2_O(MW range 678–1054) [39], while the second, smaller Hg peak possibly corresponds to [(Cys)_2_Hg] or [(Cys)_3_Hg]^-^ (MW range 442–563) or a mixture thereof [27,40]. With the 75 µM Cys mobile phase, two Hg peaks with rather similar retention times compared to those of the 50 µM Cys mobile phase were detected (peak 1: t_r_ = 1225 ± 2 s; peak 2: t_r_ = 1299 ± 3 s), but the second Hg peak was notably more intense. The utilization of the 100 µM Cys mobile phase revealed the elution of a major Hg peak (t_r_ = 1237 ± 2 s), which had a negligibly small shoulder on its long retention end. With the 200 µM Cys mobile phase, only a single but comparatively much broader Hg peak (t_r_ = 1262 s) was observed. Combined, the Hg results that were obtained with 50–200 µM Cys mobile phases revealed that Hg^2+^ is mobilized entirely from HSA, even at the lowest investigated Cys concentration of 50 µM.

With the 50 µM Cys mobile phase, an intense Cd peak eluted first (t_r_ = 925 ± 0 s), followed by three progressively smaller Cd peaks (t_r_ = 1051 ± 2 s, t_r_= 1155± 9 s, and t_r_ = 1322 ± 2 s) (Figure 2, bottom). The retention time of the smallest Cd peak was rather similar to that of the smallest Hg peak (Figure 2, dotted vertical line). The fact that the retention time of the most intense Cd peak was 38 s longer compared to that obtained for the intact HSA/Hg/Cd complex is reminiscent of previous results that were obtained on a different SEC column [11] and is rationalized by the rapid exchange of Cd between HSA and Cys during the chromatographic separation process. With the 75 µM Cys mobile phase, three Cd peaks were observed with retention times of t_r_ = 954 ± 6 s (small), t_r_ = 1069 ± 3 s (intense), and t_r_ = 1319 ± 1 s (small). The most intense Cd peak had a retention time that was rather similar to that of the second Cd peak observed with the 50 µM Cys mobile phase, suggesting a similar stoichiometric composition. Increasing the Cys mobile-phase concentration to 100 µM shifted the elution of the most intense Cd peak to a larger retention time (t_r_ = 1084 ± 3 s) compared to the 75 µM Cys mobile phase, which was followed by a less intense Cd peak (t_r_ = 1351 ± 1 s). The results for the 200 µM Cys mobile phase revealed a further shift of the most intense Cd peak to an even larger retention time (t_r_ = 1162 s) compared to the 100 µM Cys mobile phase, followed by the elution of a much smaller Cd peak (t_r_ = 1301 s). Taken together, the Cys-mediated mobilization of Cd^2+^ from HSA with 50–200 µM Cys mobile phases is in general accord with previous results in which a Cd–HSA complex was analyzed on a different SEC column with 0.5 mM Cys in a PBS buffer mobile phase [37].

The SMW thiol hCys, which is actually a metabolite, represents another biomolecule with a free thiol group that is present in the blood plasma of healthy adults at concentrations up to 12 µM [24,25]. We therefore analyzed the same HSA/Hg/Cd complex with mobile phases that contained increasing hCys concentrations (50–200 µM).

### 3.2. hCys Mobile Phase

**Figure 3:**
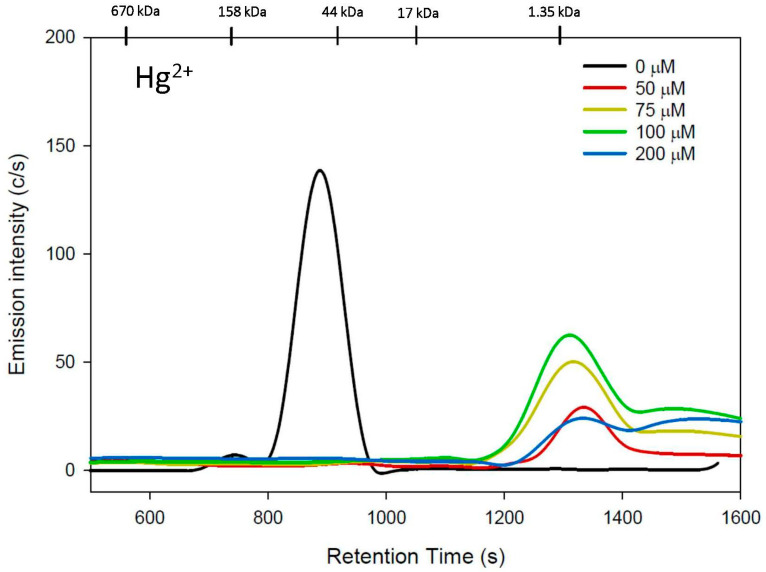
Representative Hg-specific chromatograms obtained using SEC-ICP-AES for the analysis of an HSA/Hg/Cd complex labeled with a mobile phase containing hCys (from 0 to 200 µM) in PBS buffer (pH 7.4) using a Superdex 200 Increase 10/300 GL SEC column (10 × 300 mm ID × length, 8 µm particle size, fractionation range: 600–10 kDa). Flow rate: 1.0 mL/min; injection volume: 500 µL; detector: Prodigy High-Dispersion ICP-AES of Hg at 253.652 nm. Retention times of molecular weight standards are indicated at the top. The scale of the *x*-axis was chosen to be consistent with that in Figure 1, which resulted in the remainder of the peaks not being shown. The entire peak intensities were used to calculate the peak areas shown in Table 1.

The effect of increasing hCys mobile-phase concentrations on the stability of an HSA-Hg/Cd complex is depicted in Figure 3. The obtained Hg- and Cd-specific chromatograms were used to calculate Hg and Cd recoveries of 40–93% and 5–27%, respectively (Table 1). The notably smaller Cd and Hg recoveries compared to the Cys mobile phases can be rationalized by the hCys-mediated mobilization of Hg^2+^ and Cd^2+^ from HSA, followed either by the formation of reaction product(s) with a mobile-phase component (possibly HPO_4_^2−^) or the formation of multinuclear Hg/Cd-hCys complexes that were adsorbed onto the stationary phase and were therefore not detected [41]. Owing to the poor Cd recovery, the Cd-specific chromatograms are not shown as the observed peaks were negligible.

With the 50 µM hCys mobile phase, a single and comparatively broad Hg peak eluted past the 1.35 kDa marker (t_r_ = 1337 ± 3 s), followed by significant tailing (Figure 3), which was quite different compared to the results with the corresponding Cys mobile phases (Figure 2, top). The overall peak shape implies the formation of more than one species with different sizes. Using a 75 µM hCys mobile phase produced a Hg peak (t_r_ = 1336 ± 26 s) with a rather similar elution pattern as the 50 µM hCys mobile phase, followed by a second Hg peak (t_r_ = 1504 ± 14 s). With the 100 µM hCys mobile phase, a similar Hg elution pattern was observed (Hg peak 1: t_r_ = 1329 ± 25 s), followed by a rather broad Hg peak at t_r_ = 1501 ± 16 s. Using a 200 µM hCys mobile phase, similar results were obtained, but both Hg peaks (peak 1: t_r_= 1339 ± 8 s, peak 2: 1545 ± 18 s) displayed similar relative peak intensities.

To investigate if the PBS-buffer mobile phase adversely affects the recovery, we also analyzed the HSA/Hg/Cd complex with 200 μM hCys in a 50 mM Tris-buffer mobile phase (Figure S1) and observed a Hg recovery of 71 ± 15% and a Cd recovery of only 4 ± 1% (Table 1), with the latter being even lower than that obtained for 200 μM hCys in PBS buffer. The results for Hg revealed a rather broad Hg peak (t_r_ = 1340 ± 15 s), followed by a second Hg peak (t_r_ = 1501 ± 27 s), which is consistent with the results obtained with 200 μM hCys in PBS buffer. The results obtained with both buffers strongly suggest the formation of multimeric Cd-hCys complexes, which were subsequently adsorbed onto the stationary phase.

### 3.3. GSH Mobile Phase

**Figure 4:**
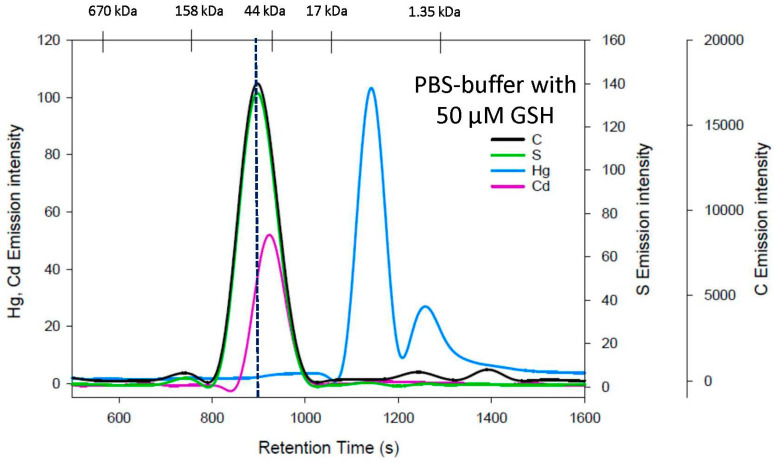
Representative C-, S-, Hg-, and Cd-specific chromatograms obtained using SEC-ICP-AES for the analysis of an HSA/Hg/Cd complex with a PBS buffer containing 50 µM GSH at pH 7.4 using a Superdex 200 Increase 10/300 GL SEC column (10 × 300 mm ID × length, 8 µm particle size, fractionation range: 600–10 kDa). Flow rate: 1.0 mL/min; injection volume: 500 µL; detector: Prodigy High-Dispersion ICP-AES multi-element detector of C at 193.091 nm, S at 180.731 nm, Hg at 253.652 nm, and Cd at 226.502 nm. Retention times of molecular weight standards are indicated at the top.

The effect of a 50 μM GSH-containing mobile phase on the stability of an HSA/Hg/Cd complex is depicted in Figure 4. Hg^2+^ was completely mobilized from HSA and eluted in the form of two complexes, with the first one being substantially larger compared to the first Hg peak that eluted with the 50 µM Cys mobile phase (difference in retention time: 115 s). In stark contrast to Hg^2+^, Cd^2+^ eluted close to HSA, as evidenced by the C and S emission lines (Figure 4).

## 4. Discussion

Chronic human exposure to toxic metal(loid)s is associated with numerous adverse health effects [42,43], but the biomolecular mechanisms that link exposure to adverse health effects are not completely understood [8,44]. Progress in enforcing more stringent environmental regulations, however, critically hinges on establishing the daily dose of a pollutant that is causally linked to the etiology of an organ-based adverse health effect (e.g., nephrotoxicity). One biological compartment in which highly relevant toxicological chemistry-related processes unfold is the systemic blood circulation, because these processes ultimately determine if the parent metal ion and/or its detoxification products impinge on toxicological target organs to determine organ-based toxicity [8]. To directly observe these processes, liquid chromatography-based approaches have shown to provide valuable insight into the formation of toxic-metal–plasma-protein complexes [45] as well as the effect of SMW thiols on the stability of the former at near-physiological conditions [13,45].

Although Hg^2+^ and Cd^2+^ are rapidly translocated from the bloodstream to their toxicological target organs [46], the detailed bioinorganic processes are not well understood [44,47]. In rats, for example, the intravenous injection with Cd^2+^ (0.4 mg Cd/kg body wt) followed by the temporal analysis of blood plasma for a RSA-Cd complex revealed its translocation to organs to be complete within 30 min [48]. A similarly rapid transport was observed when a non-nephrotoxic dose of Hg^2+^ was administered to rats, and 40% of the dose was present in the kidneys 1–3 h after exposure [49]. One way to gain insight into the involved bioinorganic processes that pertain to these translocation events is to start with plasma proteins that have distinct binding sites for Hg^2+^ and Cd^2+^. With regard to Cd^2+^, several plasma proteins have an affinity for this toxic metal ion [46,50], including HSA [51], which has two strong binding sites (log *K* = 5.3 ± 0.6) [52] and a weaker binding site. Only one of the strong binding sites, however, has been structurally characterized and shown to contain two His (67 in domain I and 247 in domain II), one Asp (249 in domain II), and one Asn (99 in domain I) [18]. In contrast, comparatively few studies have investigated the binding of Hg^2+^ to proteins in blood plasma [53], where its main binding partner appears to be the Cys-34 moiety of HSA [21]. Since Cys-34 is located in a cleft [9], Hg^2+^ is unlikely to be directly ‘handed over’ to putative biomolecular uptake mechanisms located on the surface of target organs such as the kidneys [13]. The bioinorganic processes that deliver HSA-bound Cd^2+^ and Hg^2+^ to toxicological target organs may involve other biomolecules that are also present in blood plasma, such as SMW molecules/metabolites, of which >400 are present in human plasma [54]. In fact, previous animal studies have provided direct experimental evidence that SMW thiols in blood plasma appear to play an important role in the translocation of Cd^2+^ to target organs [55], and similar results have been reported for CH_3_Hg^+^ [56].

The results that involved the utilization of an SEC-ICP-AES system to observe the effect of 50 μM Cys, hCys, or GSH dissolved in the mobile phase on the stability of an HSA/Hg/Cd complex revealed that Hg^2+^ is more effectively mobilized under these conditions compared to Cd^2+^ (Figure 2). While Hg^2+^ was mobilized from HSA to form two Hg species (possibly mononuclear and binuclear Hg complexes) [27], Cd^2+^ was mobilized to form four Cd species altogether (see red arrows in Figure 2), which may be attributed to the mobilization of Cd^2+^ from its two strong binding sites on HSA [18].

Overall, there are two reasons why these results are of toxicological relevance. Firstly, the obtained results suggest that Hg^2+^ is more ‘mobile’ at near-physiological conditions (i.e., in the presence of low µM concentrations of Cys/hCys in plasma) and therefore more easily translocated to toxicological target organs compared to Cd^2+^. These observations help to rationalize previous results from studies into the comparative uptake of Cd^2+^ and Hg^2+^ into rabbit renal cortical slices, where a faster comparative uptake of Hg^2+^ was observed [57]. Although our results were obtained from in vitro studies using a pure protein, they nevertheless provide a potential biomolecular explanation for the several-fold increased toxicity of Hg^2+^ compared to that of Cd^2+^ [58]. Secondly, the results that were obtained for Cd^2+^ imply that up to four distinct Cd species (Figure 2, bottom, red arrows) will subsequently impinge on target organs and—based on their relative organ uptake—determine toxic adverse effects therein. Taken together, these findings provide deeper insight into the dynamic bioinorganic mechanisms that deliver specific Cd^2+^ and Hg^2+^ species to their corresponding toxicological target organs [59]. Given that both Hg^2+^ and Cd^2+^ are established nephrotoxins [43,60], it is interesting to point out that both metals formed Cys complexes that eluted at about the same retention time, implying a closely related structure (Figure 2). To this end, the Cd complex (the Cd peak with the largest retention time in Figure 2) is likely to correspond to a mixture of [CdCys]^+^ and CdCys_2_ complexes, which were recently shown to be implicated in target organ uptake [32]. Future studies should be directed toward determining the structure of the detected Hg and Cd complexes, as [HgCys]^+^, [CdCys]^+^, and CdCys_2_ complexes are directly implicated in the etiology of kidney damage.

## 5. Conclusions

Ingested toxic metal species can exert organ-based toxic effects if they are taken up by their respective toxicological target organs. Since little is known about the bioinorganic processes that govern this organ uptake, we have employed an advanced liquid chromatography-based approach to gain insight. The utilization of 50 µM of Cys-, hCys-, and GSH-containing PBS-buffer mobile phases in conjunction with an SEC-ICP-AES system allowed us to observe the comparative mobilization of Hg^2+^ and Cd^2+^ from an HSA/Hg/Cd complex. All thiols completely mobilized Hg^2+^ from HSA to two Hg species; Cd^2+^ was only partially abstracted by Cys to four Cd species, while hCys likely resulted in its mobilization from HSA followed by the subsequent formation of multimeric Cd-hCys complexes that were strongly adsorbed by the stationary phase. Taken together, our results provide a feasible biomolecular explanation for the involvement of the putative Hg species Hg(Cys)_2_ and Hg(hCys)_2_ and the Cd species [CdCys]^+^ and CdCys_2_ [32] in the translocation from HSA to target organs [47] and provide a feasible explanation for the several-fold higher toxicity of Hg^2+^ in mammals compared to Cd^2+^ [58]. Furthermore, our results provide a starting point to unravel the structural basis of all observed Hg and Cd species using advanced spectroscopic tools such as X-ray absorption spectroscopy to establish the entire sequence of biomolecular processes that causally link human exposure to Hg^2+^ and Cd^2+^ with adverse organ-based effects [61,62,63,64] and possibly also with human diseases of unknown etiology [65,66,67,68].

## Figures and Tables

**Table 1:** Retention time and recovery of mercury and cadmium following injection of HSA labelled with Hg^2+^ and Cd^2+^ on a SEC-ICP-AES system using a mobile phase comprised of PBS-buffer (pH 7.4) at flow rate 1.0 mL/min and increasing concentrations of cysteine (Cys, 0 to 200 µM), and homocysteine (hCys, 0 to 200 µM) and methionine (Met, 300 µM) and glutathione (GSH, 50 µM) or Tris buffer (50 mM) and hCys (200 µM).

Mobile Phase	Concentration (µM)	Mercury		Cadmium	
		Retention Time (s) *	Total Recovery (%)	Retention Time (s) *	Total Recovery (%)
Cys	0	880 ± 2	100	887 ± 2	100
	50	1210 ± 3 ^+^ 1304 ± 2	66 ± 9	(543 ± 2) 925 ± 0 ^+^ 1051 ± 2 1155 ± 9 1322 ± 2	54 ± 6
	75	1225 ± 2 ^+^ 1299 ± 3	71 ± 17	(555 ± 3) 954 ± 6 1069 ± 3 ^+^ 1319 ± 1	75 ± 11
	100	1237 ± 2	73 ± 16	1084 ± 3 ^+^ 1351 ± 1	62 ± 9
	200	1262 ± 4	67 ± 4	1138 ± 41^+^ 1299 ± 2	51 ± 9
Met	300	744 ± 2 886 ± 1 ^+^	72 ± 21	727 ± 2 889 ± 2 ^+^	61 ± 2
h-Cys	0	743 ± 1 886 ± 2 ^+^	100	732 ± 2 888 ± 2 ^+^	100
	50	1337 ± 3	40 ± 10	538 ± 6 928 ± 2 ^+^	27 ± 8
	75	1336 ± 26 ^+^ 1504 ± 14	86 ± 21	539 ± 1 ^+^ 800 ± 135	9 ± 6
	100	1329 ± 25 ^+^ 1501 ± 16	93 ± 16	545 ± 1 ^+^ 803 ± 130	5 ± 3
	200	1358 ± 34 ^+^ 1545 ± 18 ^+^	57 ± 11	640 ± 22	20 ± 6
Tris buffer	0	709 ^#^ 858 ^+#^	100	698 ^#^ 862 ^+#^	100
	200 (hCys)	1340 ± 15 ^+^ 1501 ± 27	71 ± 15	660 ± 47	4 ± 1
GSH	0	904 ± 3	100	900 ± 1	100
	50	1143 ^+#^ 1258 ^#^	113 ^#^	924 ^+#^	50 ^#^

^*^ n = 3, ^+^ = major peak, ^#^ n = 2, average of two experiments.

## Data Availability

All data are contained within the article or supplementary material.

## Supplementary Materials

The following supporting information can be downloaded at: https://www.mdpi.com/article/10.3390/toxics11070599/s1. Figure S1: Representative C, S, Hg, Cd and Zn-specific chromatograms for the HSA/Hg/Cd complex obtained with 50 mM Tris buffer (top) and 50 mM Tris buffer with 200 μM hCys (bottom) both at pH 7.4. All experimental parameters are similar to those outlined for Figure 1, Figure 2, Figure 3 and Figure 4, with the additional emission line for Zn @ 213.865 nm.
