# Supplementary material for: More Effective Mobilization of Hg2+ from Human Serum Albumin Compared to Cd2+ by L-Cysteine at Near-Physiological Conditions
Source: Toxics. 2023 Jul 8;11(7):599. doi: 10.3390/toxics11070599 (PMC10383730; doi:10.3390/toxics11070599)
Supplement: Supplementary file 1 [file toxics-11-00599-s001.zip › toxics-2416777-supplementary.pdf]

toxics

*Supplementary materials*

# More Effective Mobilization of $Hg^{2+}$ from Human Serum Albumin Compared to $Cd^{2+}$ by L-Cysteine at Near-Physiological Conditions

**Astha Gautam and Jürgen Gailer ***

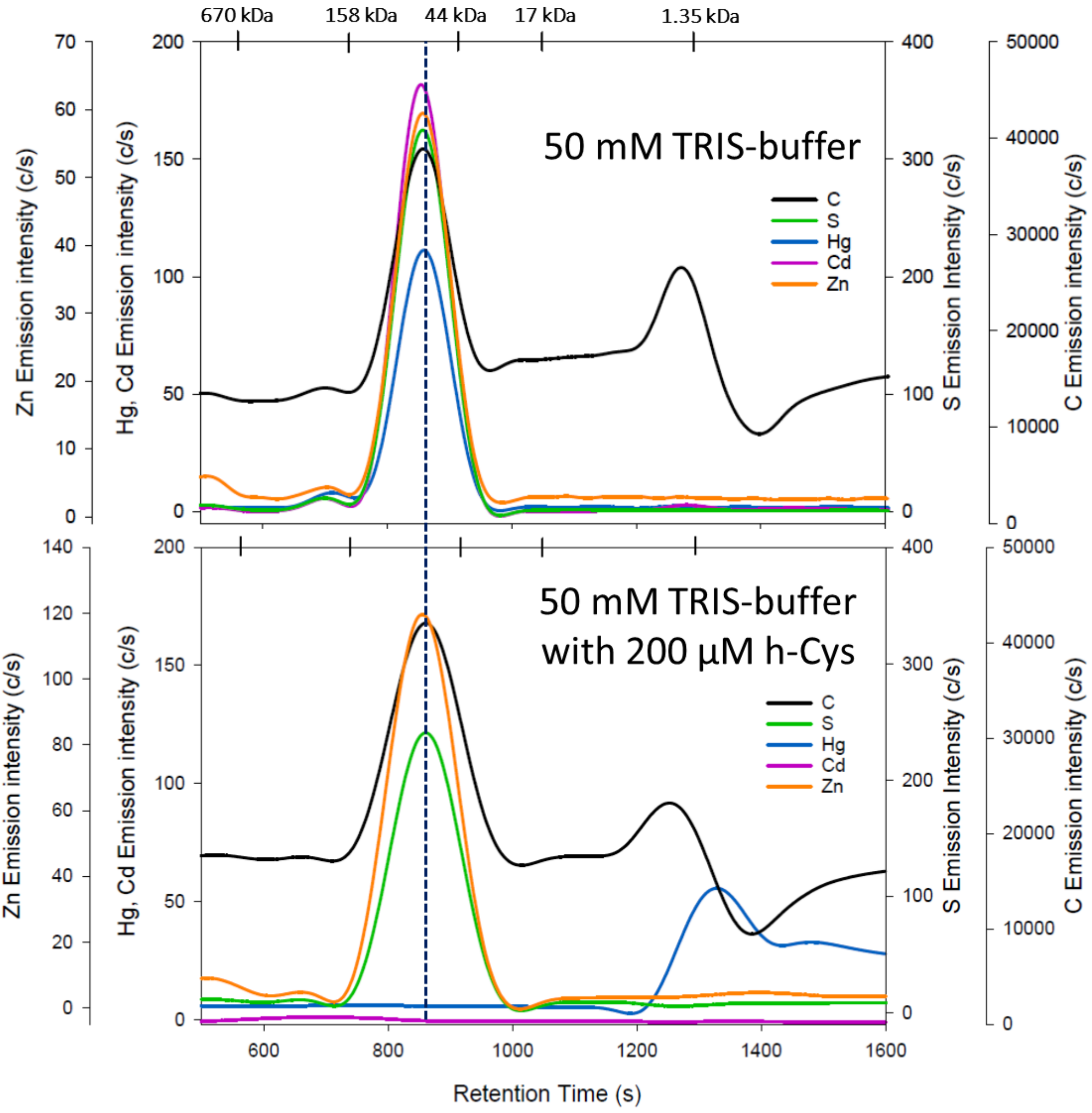


**Figure S1.** Representative C, S, Hg, Cd and Zn-specific chromatograms for the HSA/Hg/Cd complex obtained with 50 mM Tris buffer (top) and 50 mM Tris buffer with 200 µM hCys (bottom) both at pH 7.4. All experimental parameters similar to those outlined for Figs 1–4. The emission wavelength for Zn was 213.856 nm.
